# Promising trends in lung cancer care, but are we overlooking the majority?

**DOI:** 10.18632/aging.204662

**Published:** 2023-09-02

**Authors:** Bhavina Sharma, Apar Kishor Ganti

**Affiliations:** 1 Division of Oncology-Hematology, Department of Internal Medicine, University of Nebraska Medical Center, Omaha, NE 68198, USA

**Keywords:** lung cancer, aging, clinical trials

Lung cancer is the third most common cancer in the United States, after female breast cancer and prostate cancer. It accounts for more cancer-related deaths in both men and women than any other types of cancer [1]. The incidence of new lung cancer has decreased between 1999-2019, mirroring the fall in tobacco use in the past few decades. Lung cancer-related mortality has also decreased with the recent advances in screening techniques and treatment strategies. However, the incidence of lung cancer and lung cancer mortality is still disproportionately higher among older patients (65 years and older). In a recent epidemiological analysis of the US Cancer Statistics (USCS) database (2010-2017) and Surveillance, Epidemiology, and End Results (SEER-18) database, Ganti et al demonstrated that the incidence of non-small cell lung cancers (NSCLC) among older patients was more than 15 times higher than younger patients, despite a decrease in the overall incidence between 2010-2017 [2]. There was an absolute increase in the rate of new stage I NSCLC and decrease in the rate of new stage IV NSCLC among older patients, and this was attributed to the changes in the screening recommendations after 2011. Despite this positive trend in early diagnosis of NSCLC, the 5-year overall survival for older patients was noted to be lower than younger patients (25.5% vs 27.9%) with the most notable difference being in stage IV patients (4.6% vs 7.5%) [2]. The authors also noted a decrease in prevalence of NSCLC among the older patients between 2010-2016, in contrast to the increase in prevalence among younger patients. One explanation offered by the authors for this disparity was that a larger proportion of older patients was likely untreated or undertreated with single modality regimens [2].

Multiple studies have shown that older patients are more likely to be undertreated because of their chronological age, even after accounting for their comorbidities and socioeconomic status. Common reasons for this disparity are insufficient study evidence, lack of appropriate resources and support, patient factors such as socioeconomic status, as well as variations in individual physician practices and preferences. Even though the median age of lung cancer diagnosis is 71 years, and more than two-thirds of patients are older than 65 years [3], older patients are less likely to be enrolled in clinical trials. In a retrospective study evaluating eligibility criteria in cancer trials, only 3 (1%) of the 742 phase 3 randomized clinical trials included were designed for older patients [4]. Furthermore, some clinical trials may have upper age cutoff, which would limit the generalizability of these trials to older patients [4]. Older patients are especially less likely to be enrolled in early phase trials due to its vigorous assessment design, leading to scant data on dose limiting toxicities in this age group [5].

Majority of older patients receive their cancer care in the community where access to clinical trials is limited [5] Community providers may not have the appropriate resources or personnel to conduct trials on site, or they may not be aware of ongoing clinical trials in nearby academic centers [5]. For patients, particularly those who are older, it may not be feasible to pursue clinical trials that are located far from their homes due to both financial and logistical constraints. Older patients may also be reluctant to participate in clinical trials because of difficulty comprehending the trial process, concerns for complications and misinformation regarding research [5]. As such, management strategies for older patients are often extrapolated from evidence in younger and generally healthier patients; alternatively, they may be offered a less well studied option. In either case, older patients may receive less than optimal management which impacts their overall prognosis.

With the majority of lung cancer patients being older than 65, it is imperative that actions are taken to encourage and facilitate clinical trials among older patients. Creative and adaptive clinical trial designs with stepwise dosing approach should be encouraged [5]. Instead of relying on chronological age as an eligibility criterion, comprehensive geriatric assessment tools that includes functional, cognitive, nutritional, frailty indices should be employed to evaluate the vigor of the older patients [6]. In a cluster randomized trial by Mohile at al, older patients (70 years and older) with solid tumor or lymphoma who received tailored geriatric assessment were found to have less treatment-related toxicity (51% vs 71%) and fewer falls over 3 months (12% vs 21%) [7]. Apart from disease specific outcomes, function specific endpoints focused in the above-mentioned areas should be promoted. Older patients should be encouraged to participate in early phase trials to help establish the pharmacokinetic and pharmacodynamic parameters that are appropriate for them [5].

To facilitate recruitment of a more representative group, academic institutions should be incentivized to collaborate with private practices to conduct clinical trials in the community [5]. Social media, mainstream media channels as well as community outreach programs may be useful to raise awareness among providers and the public. Education workshops with special focus on geriatric patients should be offered to relevant providers. More importantly, the clinical trial enrollment process should be streamlined and made simpler. Screening studies can be combined to reduce the number of total visits. The clinical trial-related documents should be kept short and concise and written in easily understandable language [8]. Older patients who are willing to participate in clinical trials should be offered additional support, psychosocial as well as logistical, to encourage their continued participation.

Although there is now increasing effort and guidance by major cancer societies and regulatory groups to increase inclusion of older patients, better conscious collaboration between the stakeholders is necessary for effective implementation of the strategies discussed and to enhance enrollment and retention of older cancer patients.
